# Trends in healthcare expenditure among people living with HIV/AIDS in the United States: evidence from 10 Years of nationally representative data

**DOI:** 10.1186/s12939-017-0683-y

**Published:** 2017-10-27

**Authors:** Tiarney D. Ritchwood, Kinfe G. Bishu, Leonard E. Egede

**Affiliations:** 1 Department of Public Health Sciences, Medical University of South Carolina, Charleston, SC USA; 2 Center for Health Disparities Research, Department of Medicine, Medical University of South Carolina, Charleston, SC USA; 3 Department of Medicine, Division of General Internal Medicine and Geriatrics, Medical University of South Carolina, Charleston, SC USA; 4 Center for Patient Care and Outcomes Research (PCOR), Medical College of Wisconsin, 8701 Watertown Plank Road, Milwaukee, WI 53226 USA; 5 Division of General Internal Medicine, Medical College of Wisconsin, Milwaukee, WI USA

**Keywords:** HIV, Cost, Medical expenditure panel survey, Chronic illness

## Abstract

**Background:**

While previous studies have examined HIV cost expenditures within the United States, the majority of these studies focused on data collected prior to or shortly after the advent and uptake of antiretroviral therapy, focused only on a short time frame, or did not provide cost comparisons between HIV/AIDS and other chronic conditions. It is critical that researchers provide accurate and updated information regarding the costs of HIV care to assist key stakeholders with economic planning, policy development, and resource allocation.

**Methods:**

We used data from the Medical Expenditure Panel Survey-Household Component for the years 2002–2011, which represents a nationally representative U.S. civilian non-institutionalized population. Using generalized linear modeling, we estimated the adjusted direct medical expenditures by HIV/AIDS status after controlling for confounding factors.

**Results:**

Data were from 342,732 people living with HIV/AIDS. After adjusting for socio-demographic factors, comorbidities and time trend covariates, the total direct expenditures for HIV/AIDS was $31,147 (95% CI $23,645–$38,648) or 800–900% higher when compared to those without HIV/AIDS (i.e., diabetes, stroke, and cardiovascular disease). Based on the adjusted mean, the aggregate cost of HIV/AIDS was approximately $10.7 billion higher than the costs for those without HIV/AIDS.

**Conclusions:**

Our estimates of cost expenditures associated with HIV care over a 10-year period show a financial burden that exceeds previous estimates of direct medical costs. There is a strong need for investment in combination prevention and intervention programs, as they have the potential to reduce HIV transmission, and facilitate longer and healthier living thereby reducing the economic burden of HIV/AIDS.

## Background

In 2013, almost 7000 people in the United States died due to complications from HIV/AIDS [1]. Though this number is less than half of that reported a decade earlier, HIV/AIDS is still among the leading causes of death within the United States and worldwide [1]. The introduction of highly active antiretroviral therapy (HAART) was largely responsible for the substantial declines in HIV-related morbidity and mortality [2]. In fact, the number of new annual infections has been reduced by more than 60% since 1985 [1]. With approximately 1.2 million people living with HIV/AIDS (PLWHA) [1], there have been increasing concerns about the costs associated with lifetime treatment of the infection, including costs associated with accessing medical care, HIV medications, and costs for treating co-morbid conditions. Though previous studies have projected lower healthcare costs due to the availability of HAART and fewer hospitalizations due to AIDS-defining illnesses, more recent studies have suggested that, for a proportion of PLWHA, frequent and lengthy hospitalizations are common, which are associated with high personal costs and healthcare expenditures [3, 4].

The cost of HIV care in the United States is a critical issue considering that there are approximately 1.2 million PLWHA. A recent study estimated that the lifetime medical costs for an individual who becomes infected with HIV at age 35 was $326,500, with 60% of those expenses attributable to the costs of antiretroviral medication [5]. This estimate was 3.3 times higher than the lifetime costs of medical care for high-risk individuals who remain uninfected and include the medical costs related to treating both HIV and non-HIV-related conditions. In addition to costs incurred due to longer lifespans, the rates of new infections are on the rise within vulnerable populations, particularly among young, Latino and African American men who have sex with men (MSM) between the ages of 13 and 24 years [1]. Between 2005 and 2015, HIV infections rose by 87% among young, African American MSM; however, more recent figures suggests that the dramatic increase is beginning to level off [1]. Among Latino MSM, new infection rates rose by 24% during the same period [1]. These figures are concerning, as members of vulnerable groups are less likely to engage in outpatient care and more likely to seek in-hospital care, which is associated with higher cost expenditures [4]. Additionally, in 2002, the costs associated with new HIV cases in the United States were estimated to be $6.7 billion in direct medical costs and $29.7 billion in indirect costs that result from productivity losses, which describes lost economic opportunities as a result of illness, disability, or premature death [6]. These estimates do not include economic losses that occur as a result of caregiving responsibilities or morbidity, which suggests that the true economic burden may far exceed the estimated costs [6]. The costs for lifetime treatment based upon the number of new diagnoses were estimated to be $16.6 billion in 2009 [7]. Longer lifespans and the over-representation of members from vulnerable groups living with the virus have important implications for healthcare systems, particularly as it relates to resource utilization and allocation, and cost expenditures [2].

Though there have been a number of studies that have examined the costs of HIV care within the United States, the majority of these studies focused on data collected prior to or shortly after the advent and uptake of HAART, were limited by a focus on a short time frame, or have not provided cost comparisons between HIV/AIDS and other chronic conditions [2, 6, 8–11]. Since the early years of the HIV epidemic, researchers were tasked with providing accurate and updated information regarding the costs of HIV care to assist key stakeholders and decision-makers with economic planning, policy development and adjustments, economic evaluations of interventions focused on treatment costs, and resource allocation [5]. Therefore, the current study uses data from a nationally representative sample to assess direct healthcare expenditures for PLWHA from 2002 to 2011 in the United States when compared to those without HIV, but suffering from other chronic conditions.

## Methods

### Data source and population

We analyzed data from participants in the Medical Expenditure Panel Survey-Household Component (MEPS-HC) for the years 2002–2011 without restrictions on age. MEPS is a survey of a nationally representative, non-institutionalized, and U.S. civilian-based population administered by the Agency for Healthcare Research and Quality (AHRQ) [12]. The AHRQ validates MEPS data by administering a variety of quality assurance measures and compares MEPS numbers with data from other sources, such as the Census Bureau and National Health Interview Survey (NHIS). MEPS obtains information on participants’ use of medical care and their medical spending, as well as information on demographics, socioeconomics, and health conditions. Medical expenditures are defined as the payments that health care providers receive from all payers (including insurance providers, survey respondents, and other sources) [13]. Data for the MEPS-HC is obtained by means of self-report.

Medical and financial information is obtained from medical records, reports from physicians and home health care providers, and pharmacies. Diagnoses coded according to ICD-9-CM (International Classification of Disease, Ninth Revision, Clinical Modification) are also collected as part of the Medical Provider Component of the MEPS. The medical conditions and procedures reported by the MEPS-HC related to HIV/AIDS were recorded by the interviewer as verbatim text and then converted by professional coders to ICD-9-CM codes. The error rate for any coder did not exceed 2.5% on verification. To protect the confidentiality of respondents, fully specified ICD- 9-CM codes were collapsed to three digits [12].

Individuals with HIV/AIDS were extracted from MEPS-HC medical condition files using ICD-9-CM codes at the person level. For each year, we merged data from the MEPS-HC medical condition files and the full-year consolidated files using the unique person identifier (DUPERSID) on a one-to-one match. To ensure a sufficient sample size and robust estimation for our analysis, we pooled the 10-year MEPS data due to their common variance structure, which is necessary to ensure comparability of our variables within the complex sample design. We adjusted the analytic sampling weight variable by dividing it by the number of years being pooled. The sum of these adjusted weights represents the average annual population size for the pooled period. Estimates of totals are based on adjusted weights and reflect an “average annual” basis rather than the entire pooled period. The MEPS has a complex design consisting of clustering, stratification, and multistage and disproportionate sampling with oversampling of minorities [13]. The 2002–2011 direct healthcare costs were adjusted to the value of the US dollar in 2014 using the consumers price index obtained from the Bureau of Labor Statistics (BLS) (http://data.bls.gov/cgi-bin/cpicalc.pl).

### Measures

#### Outcomes

Dependent variables in this study are the direct costs for total healthcare expenditures, defined as the sum of direct payments for care provided during the year, including out-of-pocket payments and payments by private insurance, Medicaid, Medicare and other sources [13]. The total medical healthcare expenditure is a sum of office-based medical provider expenditure, hospital outpatient expenditure, emergency room expenditure, inpatient hospital (including zero night stays) expenditure, prescription medicine expenditure, dental expenditure, home health care expenditure and other medical expenses [13].

#### Primary independent variable

The primary independent variable is people with HIV/AIDS. We used ICD-9-CM codes as defined in the MEPS to represent disease conditions. We used ICD-9-CM diagnostic codes of 42 and V08 to identify patients with HIV/AIDS.

#### Covariates

All covariates used for analysis were based on self-report. Co-morbidities: Binary indicators of co-morbidities were based on a positive response to a question “Have you ever been diagnosed with diabetes, hypertension, stroke, emphysema, joint pain, arthritis and asthma?” Cardiovascular Disease (CVD) indicates a positive response to a question “Have you ever been diagnosed with coronary heart disease, angina, myocardial infarction, or another heart disease?” A binary variable of comorbidity showed if patients had at least one comorbidity. Race/ethnic group was categorized into non-Hispanic Whites (NHW) vs. Others (Non-Hispanic Blacks (NHB), Hispanics, other races). A binary indicator of education was coded as less than high school (≤ grade 11) vs. high school or more. Marital status was coded as married vs. non-married (Widowed/Divorced/separated, or never married). Sex was coded as female vs. male and age was coded as a continuous variable in years. Census region was coded as: South vs. Other regions (Northeast, Midwest, and West). Metropolitan Statistical Areas (MSA) was coded yes =1 as of the end of the year. Health insurance was coded into insured vs. uninsured at all times in the year. The income level was defined as a percentage of the poverty level and grouped into two categories: poor (<125%) vs. low income (125%) or more. Calendar year was grouped into three consecutive years, 2002/04, 2005/07, and 2008/11 for the pooled data.

### Analyses

The baseline characteristics of patients were compared by HIV/AIDS status and presented as percentages for categorical variables. We tested differences between values using χ^2^ tests. We estimated the unadjusted mean for direct healthcare expenditures for individuals by HIV/AIDS status using the test post-estimation command. We then used generalized linear modeling (GLM) to estimate the adjusted direct medical expenditures by HIV/AIDS status after controlling for confounding factors. The GLM has been widely employed in situations where, due to large number of non-users of health services, there are excess zeros in cost data and the assumption of normality of the error term is not satisfied [14]. The model addresses the zero concentration as well as the positive skewness of expenditures [15] and allows users to calculate incremental effects and standard errors [16]. The use of GLM has an advantage over log OLS, as it relaxes the normality and homoscedasticity assumptions and avoids bias associated with the transformation of raw scaled scores [16]. While a two-part econometric model (family gamma, log link) can model non-negative values and heavily-skewed cost distribution data, we chose GLM because the two-part model failed to provide estimates due to the small percentage of individuals with HIV/AIDS diagnoses (0.14%) in our analysis. To control for confounding, socio-demographic factors including age, sex, race, marital status, education, health insurance, metropolitan statistical area status, region, and income level, comorbidities and time trend were included in the model. We used Pearson’s χ^2^ tests for Table 1, adjusted Wald F test for Table 2 and z-test for Table 3 to measure significance level.

**Table 1:** Weighted sample demographics by HIV/AIDS status among US population 2002-2011

Variables	All (%)	HIV (%)	No-HIV (%)	*p-value
N	244,124,177	342,732 (0.14%)	243,781,444 (99.86%)	
Gender				
Male	46.9	80.7	46.8	<0.001*
Female	53.2	19.3	53.2	
Race/ethnicity				
Non-Hispanic White	69.6	46.4	69.7	<0.001*
Other race	30.4	53.6	30.3	
Marital status				
Married	53.5	9.5	53.6	<0.001*
Non-married	46.5	90.5	46.4	
Education category				
<High School	31.3	23.0	31.3	<0.001*
High School or more	68.8	77.0	68.7	
Insurance				
Insured	89.9	91.9	89.9	0.302
Uninsured	10.1	8.1	10.1	
Metropolitan statistical status				
Urban	83.0	95.6	83.0	<0.001*
Rural	17.0	4.4	17.0	
Census region				
South	36.0	35.6	36.0	0.925
Other regions	64.0	64.4	64.0	
Income category				
Poor income	16.8	38.8	16.8	<0.001*
Low income or more	83.2	61.2	83.2	
Chronic conditions				
Diabetes	8.3	11.3	8.3	0.154
Hypertension	32.8	32.9	32.8	0.961
CVD	13.6	13.7	13.6	0.978
Stroke	3.5	3.6	3.4	0.893
Emphysema	2.1	1.9	2.1	0.806
Joint pain	37.8	39.6	37.8	0.634
Arthritis	26.0	25.0	26.0	0.774
Asthma	11.0	16.2	11.0	0.023*
Comorbidity	51.8	68.5	51.8	<0.001*
Year category				
Year 2002/04	29.2	26.4	29.2	0.086
Year 2005/07	29.7	31.0	29.7	
Year 2008/11	41.1	42.6	41.1	

Note: *denotes that *p* < 0.05 for each pair of variables for which association was measured

**Table 2:** Unadjusted means of total healthcare and healthcare service expenditures by HIV status

	HIV		No HIV		*p*-value
	Mean ($)	95% CI	Mean ($)	95% CI	
Total Cost					
2002/04	22,544	15,901–29,187	4,407	4,243–4,571	<0.001**
2005/07	27,247	16,548–37,946	4,760	4,620–4,900	<0.001**
2008/11	29,329	21,817–36,840	5,013	4,877–5,150	<0.001**
Pooled sample	26,893	21,844–31,943	4,761	4,669–4,854	<0.001**
Inpatient					
2002/04	3,874	575 –7,173	1,290	1,179–1,401	0.124
2005/07	4,698	1,115–8,281	1,331	1,250 –1,412	0.065
2008/11	4,162	290–8,034	1,358	1,275–1,441	0.154
Pooled sample	4,252	2,028–6,476	1,330	1,277–1,384	0.010*
Office-based					
2002/04	2,112	1,407–2,817	1,010	980–1,040	0.001**
2005/07	2,410	929–3,891	1,156	1,112–1,199	0.095
2008/11	3,214	2,012–4,415	1,249	1,213–1,285	0.001**
Pooled sample	2,674	1,945–3,403	1,152	1,129–1,175	<0.001**
Medications					
2002/04	13,833	10,194–17,472	907	877–937	<0.001**
2005/07	18,573	8,708–28,438	1,045	1,008–1,083	<0.001**
2008/11	19,820	15,306–24,334	1,116	1,067–1,166	<0.001**
Pooled sample	17,854	14,061–21,646	1,034	1,007–1,061	<0.001**
Outpatient					
2002/04	446	419–473	966	174–1,758	0.197
2005/07	320	81–558	449	422–476	0.288
2008/11	481	179–783	474	444–504	0.959
Pooled sample	559	321–798	458	440–476	0.407
Emergency Room (ER)					
2002/04	451	-38 –940	162	155–170	0.247
2005/07	278	45–511	169	161–177	0.354
2008/11	192	65–320	205	195–215	0.841
Pooled sample	287	104–470	182	177–187	0.260
Home Health					
2002/04	929	4–1,855	152	113–191	0.100
2005/07	444	77–810	168	120–217	0.145
2008/11	956	-236–2,149	179	149–209	0.200
Pooled sample	790	215–1,365	168	140–195	0.034*
Dental					
2002/04	249	121–378	339	328–350	0.171
2005/07	342	136–548	341	329–353	0.992
2008/11	390	193–588	338	325–351	0.597
Pooled sample	338	226–451	339	331–347	0.987
Other					
2002/04	126	91–103	126	70–182	0.309
2005/07	97	92–103	179	105–253	0.029*
2008/11	110	49–171	91	86–95	0.531
Pooled sample	94	91–98	136	98–173	0.030*

Note: Inpatient=Hospital Inpatient; Outpatient=Hospital Outpatient; Total healthcare expenditure= the sum of direct payments for care provided during the year, including out-of-pocket payments and payments by private insurance, Medicaid, Medicare and other sources; Total medical healthcare expenditure= sum of office-based medical provider expenditure, hospital outpatient expenditure, emergency room expenditure, inpatient hospital (including zero night stays) expenditure, prescription medicine expenditure, dental expenditure, home health care expenditure and other medical expenses; **p ≤ 0.01; *p ≤ 0.05

**Table 3:** Generalized linear model: Incremental healthcare expenditures by HIV status among US individuals accounting for relevant covariates (adjusted to 2014 dollars)

Variables	Incremental Cost	95% CI	*P*-value
Primary Independent Variable			
HIV (vs. no HIV)	31,147**	23,645 – 38,648	<0.001
Covariates			
Female (vs. Male)	1,419**	1,181 – 1,657	<0.001
Other race (vs. non-Hispanic White)	-866**	-1,115 – -617	<0.001
Non-married (vs. Married)	-380**	-573 – -187	<0.001
Age (in years)	63**	55–71	<0.001
High school or more (vs. <High School)	619**	360 – 879	<0.001
Uninsured (vs. Insured)	-3,642**	-3,809 – -3,475	<0.001
Urban (vs. Rural)	308*	58 – 557	0.015
Other regions (vs. South)	377**	158 – 595	0.001
Low income or more (vs. Poor income)	-1,221**	-1,601 – -841	<0.001
Diabetes (vs. no Diabetes)	2,971**	2,640–3,301	<0.001
Hypertension (vs. no Hypertension)	1,249**	1,052 – 1,446	<0.001
CVD (vs. no CVD)	3,364**	3,043 – 3,685	<0.001
Stroke (vs. no Stroke)	2,945**	2,462 – 3,446	<0.001
Emphysema (vs. no Emphysema)	2,294**	1,732 – 2,855	<0.001
Joint pain (vs. no Joint Pain)	1,032**	827 – 1,238	<0.001
Arthritis (vs. no Arthritis)	1,577*	1,350 – 1,805	<0.001
Asthma (vs. no Asthma)	1,405***	974 – 1,836	<0.001
Comorbidity (vs. no Comorbidity)	378*	35–721	0.031
Year 2005/07 (vs. Year 2002/04)	356*	73 – 640	0.014
Year 2008/11 (vs. Year 2002/04)	367*	87 – 647	0.010

Note: ***p < 0.001; **p ≤ 0.01; *p ≤ 0.05; Primary outcome variable in this model is total health care expenditures and the incremental (marginal) effect represents the primary independent variable (HIV/AIDS) and other covariates

The Generalized Linear Model (GLM) method is efficient in modeling cost data with the appropriate choice of the variance function. The modified Park test can be used as a diagnostic test to examine the model fit [16]. The gamma model is used for data situations in which the response can take only values greater than or equal to zero [17]. Multicollinearity was assessed for predictors of the GLM taking into account the complex survey design.

For all the analyses, we accounted for the complex sampling design of MEPS by using sampling weight, variance estimation stratum and primary sampling unit (clustering) in order to extrapolate the estimates to a U.S civilian non-institutionalized population. A *p*-value <0.05 was considered statistically significant. All analyses were performed using STATA 14.

## Results

### Population characteristics

The characteristics of US population with and without HIV/AIDS during the 2002–2011 period are shown in Table 1. Of the weighted 244,124,177 people in the U.S. population, 342,732 (0.14%) had HIV/AIDS. HIV/AIDS was more frequent among those who: identified as male, were members of other racial groups, were non-married, had at least completed high school, were urban dwellers, poor income earners and reported comorbid conditions. While HIV/AIDS prevalence increased in 2005/2007 and 2008/2011, these changes were not statistically significant.

### Unadjusted cost differences between individuals with and without HIV/AIDS

**Fig. 1:**
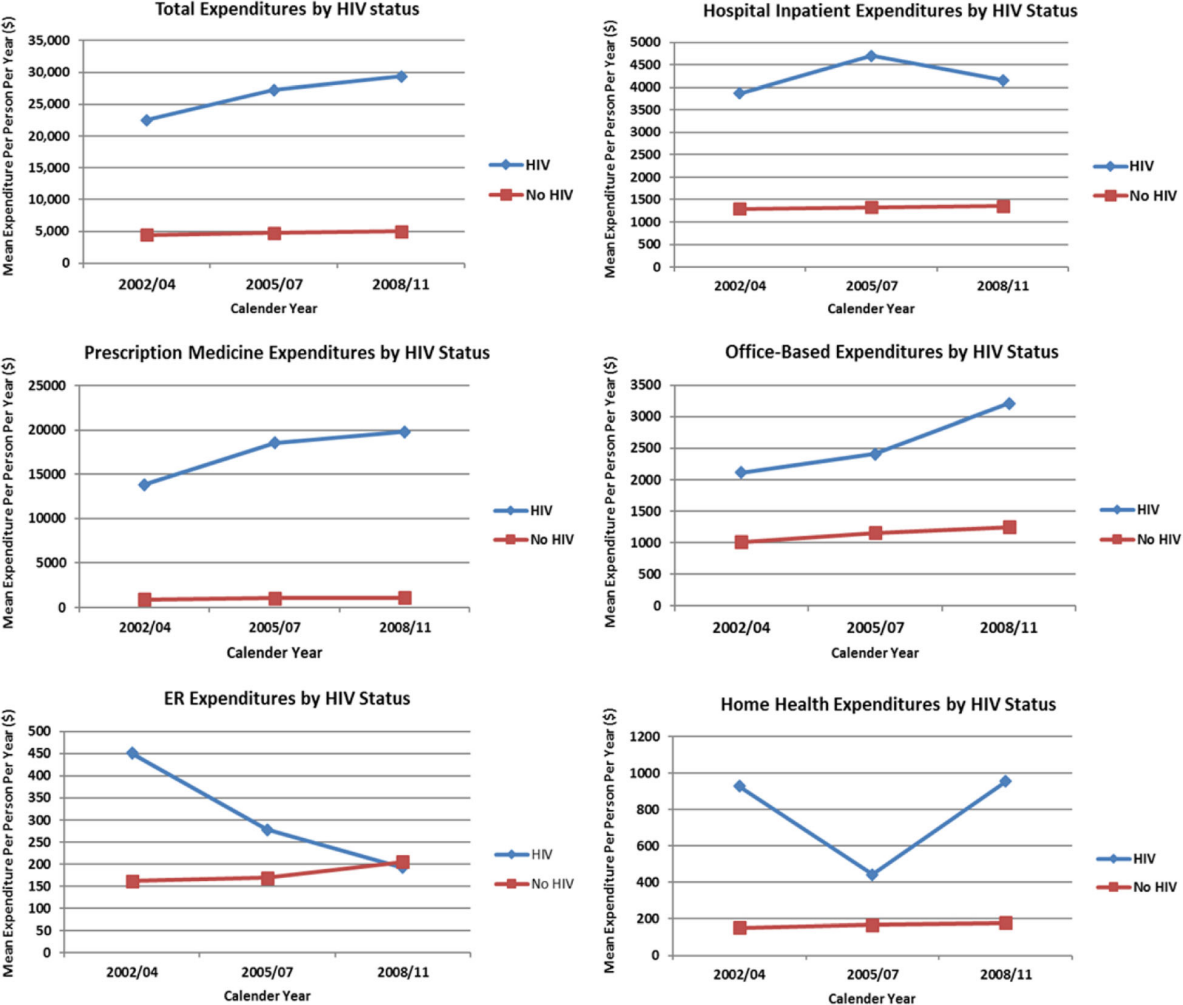
Trends in total direct expenditures and healthcare services by HIV status

The total mean unadjusted direct expenditures for individuals with HIV/AIDS consistently increased from $22,544 (95%CI: 15,901–29,187) in 2002/2004 to $29,329 (95%CI: 21,817–36,840) in 2008/2011 (Table 2 and Fig. 1). Relative to the direct expenditures for individuals without HIV/AIDS ($4761 [$4669–$4854]), PLWHA had nearly six times higher unadjusted mean expenditure ($26,893[$21,844–$31,943]) over the 10-year pooled information period (Table 2). PLWHA had increased inpatient hospital expenditures from 2002/ 2004 ($3874 [$575–$7171]) to 2005/08 ($4698 [$1115–$8281]) but then decreased in 2008/2011 ($4162 [$290–$8034]). PLWHA had continuously increased prescription medicine expenditures from 2002/2004 ($13,833 [$10,194–$17,472]) through 2008/2011 ($19,820 [$15,306–$24,334]). Prescription medicine expenditures ($17,854) of HIV/AIDS accounts for the largest proportion (66%) of the total medical expenditure. Trends in office-based expenditures for PLWHA were similar, with costs increasing continuously from 2002/2004 ($2112 [$1407–$2817]) through 2008/2011 ($3214 [$2012–$4415]).

### Adjusted incremental cost differences for PLWHA

The results of the adjusted GLM on incremental costs associated with HIV/AIDS controlling for socio-demographic factors, comorbidities and time trends are shown in Table 3. The results of the modified Park test verified the use of a gamma distribution with a log link as the best–fitting GLM to get consistent estimation of coefficients and marginal effects of medical expenditure [17]. The variance inflation factor (VIF) for all predictors used in the model indicated there was no multicollinearity. After adjusting for socio-demographic factors, comorbidities and time trend covariates, the adjusted incremental cost of total direct expenditures for HIV/AIDS increased by $31,147 (95% CI $23,645–$38,648) when compared to those without HIV/AIDS (Table 3). Being female, more educated, and having comorbidities were significantly associated with higher incremental cost compared with their counterparts. Each one year increase in age was associated with $63 higher incremental expenditures. Compared with the South, residents in other regions had $377 higher incremental expenditures. Non-White race/ethnic group, non-married and the uninsured had significantly lower incremental costs compared with their counterparts. Compared with 2002/2004, incremental expenditures were significantly higher by $356 in 2004/2005 and $367 in 2008/2011. HIV/AIDS was associated with 800–900% higher incremental expenditures relative to other high cost chronic conditions (i.e., diabetes, stroke, and CVD).

### Estimated US burden of HIV/AIDS

Finally, we extrapolated the aggregate cost to the US population for PLWHA and those without during 2002–2011. Based on the adjusted mean, the aggregate incremental cost of HIV/AIDS was approximately $10.7 billion higher than the costs for those without HIV/AIDS.

## Discussion

Despite the decline in AIDS-related morbidity and mortality, the financial burden of HIV resulting from longer lifespans and the rise of new infections among vulnerable groups in the United States is considerable. The purpose of the current study was to provide updated estimates of direct healthcare expenditures for PLWHA using data from a nationally representative sample of patients receiving care from diverse provider sites. Compared to individuals without HIV, PLWHA were more likely to be members of racial minority groups, unmarried, poor, less educated, and report comorbid chronic conditions. Our results support previous studies that have highlighted concerns regarding the ‘browning’ of HIV, as PLWHA are more likely to face social and structural challenges that contribute to higher rates of infection and subsequently, a higher financial burden [6]. Moreover, members of vulnerable populations may be more likely to enter HIV care at more advanced disease stages, which has been associated with greater healthcare utilization and thus higher costs [2, 18].

To our knowledge, this is the first study to compare the financial burden of PLWHA and those living with other chronic conditions. We found that PLWHA had significantly higher direct healthcare expenditures between 2002 and 2011 than their counterparts; they reported higher costs of prescription medications, and higher utilization of inpatient, office-based, and home health services. Moreover, our analyses indicated that PLWHA incur incremental costs that are approximately $10.7 billion**,** or 800–900%, higher than their counterparts without HIV but living with other chronic conditions (i.e., CVD, diabetes, and stroke). For PLWHA, the largest proportion of cost expenditures resulted from prescription medications, which increased steadily during the study period to account for 66% of total expenditures, costing $17,854 in 2008/2011, which is approximately $4000 more than medication-related expenditures in 2002/2004. Our results are consistent with earlier studies finding that costs of prescription medications are increasing over time and aside from mortality-related productivity loss, represents the most burdensome cost to PLWHA and the healthcare system [5, 19]. As such, changes in HIV cost expenditures are largely dependent upon fluctuations in medication costs, which may be expected to decrease as expiring patents make way for generic options [9, 20].

Our overall annual, per-person cost estimate ($31,147) was substantially higher than those of earlier studies (mean total costs between $18,640 and $20,300) [4, 7, 19]. These higher costs are likely due to the wide availability of HAART and longer life expectancies, which require people to take HAART over longer periods of time, contributing to higher direct cost expenditures [4, 5]. Previous research, however, has suggested that universal uptake of HAART could lead to lower indirect cost expenditures, as better health among PLWHA and longer lifespans could lead to greater economic productivity [6]. For those with other chronic conditions, the largest proportion of cost expenditures resulted from inpatient and office-based fees, followed by prescription medication costs. However, the costs of prescription medications for PLWHA were almost 5 times higher than the top three cost expenditures for those without HIV, combined. The results of this study suggest that HIV/AIDS remains a significant concern for the United States and requires additional resources and research to identify ways to reduce HIV care costs, yet improve the quality of care. Considering the needs of those most vulnerable to infection, access to HAART may become increasingly difficult for individuals if policies and allocation of resources are not improved. There is a strong need for financial investment and socio-structural interventions to reduce the financial burden on those most impacted by HIV in order to facilitate longer and healthier lives [5].

The current study has several major advantages over previous studies, including: 1) an examination of cost trends using data from a nationally representative survey; 2) an analysis of cost expenditures using a variety of categories, including prescription medications, inpatient services, office-based care, dental treatment, emergency room visits and home health services; 3) the utilization of a novel methodology that enables incremental cost estimates; 4) accounting for multiple comorbidities in an attempt to isolate the effect of HIV/AIDS; 5) the inclusion of unadjusted cost estimates to provide further insight into the actual costs amassed by PLWHA; and 6) using pooled analyses that capture cost trends over a 10-year period of time.

Though this study has a number of strengths, our results are also subject to several limitations. First, HIV/AIDS status was determined by means of self-report and was not confirmed by the use of laboratory tests. As a result, it is possible that HIV status was under-reported, considering that a large proportion of PLWHA are unaware of their HIV status ^1^. However, people unaware of their HIV status will not be accessing HIV care so will likely have few, if any, incurred costs. This method, however, has been commonly used in the cost estimation literature and for those who are aware of their status, reporting is likely to be accurate [21]. Second, due to limitations of secondary data analyses, we are unable to access indirect costs associated with HIV, including loss of productivity and a decreased quality of life. As a result, the current findings cannot estimate the indirect costs associated with accessing and utilizing HIV care. Lastly, the current data, though sensitive to change over time, is pooled and therefore cannot be interpreted as representative of longitudinal trends.

## Conclusions

Our estimates of cost expenditures associated with HIV care over a 10-year period show a financial burden that exceeds previous estimates of direct medical costs. However, these results do not consider cost expenditures that result from indirect sources, such as productivity loss and early disability and mortality. Though PLWHA are likely to incur greater healthcare costs over time due to prescription medication, the clinical benefits to the person, as well as the larger community, may be substantial and could lead to fewer viral transmissions, saving approximately $230,000 for each averted infection [5]. There is a strong need for investment in combination prevention and intervention programs, as they have the potential to reduce HIV transmission, and facilitate longer and healthier living thereby reducing the economic burden of HIV/AIDS. Future research should consider how socio-structural factors interact with demographic characteristics to influence HIV cost expenditures. With changing political climates, the outcomes of PLWHA might be greatly impacted. The Affordable Care Act (ACA), for example, offered substantial benefits to PLWHA, including: 1) access to private insurance coverage; 2) expanded Medicaid eligibility based upon income and residency; 3) elimination of annual and lifetime benefit costs; and 4) elimination of insurance companies abilities to deny coverage based upon pre-existing conditions [22]. Recent efforts to repeal the ACA could lead to less healthcare coverage for PLWHA, especially childless adults and those at greatest risk for transmitting the virus, leading to increases in new infections and higher healthcare costs. It is therefore critical that public health researchers, advocates, and physicians partner with lawmakers to ensure that PLWHA who are most vulnerable to poor health outcomes have equal access to healthcare.
